# A Medical Law for the Masses

**Published:** 1872-11

**Authors:** 


### Editorial.

### A Medical Law for the Masses.

Most of our medical legislation for the past years has been in the interest of some medical denomination, and been urged upon the legislature for privilege or protection. We have now to propose a measure in the interest of the people, and one which we believe all schools of medicine will alike favor. It is well known that in the State of New York the practice of medicine and surgery is open to all; it is a broad field into which men of all classes and all occupations may enter and find employment ; nothing of special fitness is required. The shopman and shoemaker, the hoseboy, nurse or barber,—all may at pleasure assume to direct the care of the sick and administer medicines for their kill or cure. This ought not so to be ; the care of the sick should not be left in wholly uneducated hands : some conditions of qualification should be imposed, even if inadequate and unsatisfactory. Our law-makers have no moral right to leave the people thus unprotected in an interest which oftentimes involves life. It may be thought that the selection of care when sick may safely be left to individual judgment ; but, alas ! this is not so. The citizens of New York are practiced upon by harpies and charlatans, who say they are physicians, and profess most wonderful knowledge of disease and means of cure, while in reality they are not physicians at all, have never learned any of the collateral branches of medicine, and know nothing in any of the departments of medicine ; we mean they are not physicians in any sense, not educated in any system, belong to no school, and have no claim to the name of physicians.

We ask for the community protection from this class of practitioners, and we invite the aid and co-operation of physicians of all denominations to obtain a law which shall prevent the practice of medicine by those who have not diplomas from incorporated schools of medicine, or membership in some recognized medical society. In this we believe we shall receive the support of all right-minded men of all persuasions, and to obtain this it may be well to leave every other restriction which we might desire to see placed upon quackery and be satisfied for the present with a measure which cannot be opposed upon any rational grounds of objection.

We hope that Erie County Medical Society will take early steps in this matter, and bring the subject to the attention of the State Medical Society and State Legislature at the approaching sessions. Influential and earnest men in Buffalo stand ready to second the effort, and it is to be hoped that New York will receive the protection which has been for some time enjoyed in Pennsylvania, Ohio, Wisconsin, and indeed most other States.

